# Perioperative hemodynamic optimization: from guidelines to implementation—an experts’ opinion paper

**DOI:** 10.1186/s13613-021-00845-1

**Published:** 2021-04-14

**Authors:** Jean-Luc Fellahi, Emmanuel Futier, Camille Vaisse, Olivier Collange, Olivier Huet, Jerôme Loriau, Etienne Gayat, Benoit Tavernier, Matthieu Biais, Karim Asehnoune, Bernard Cholley, Dan Longrois

**Affiliations:** 1 Service D’Anesthésie-Réanimation, Hôpital Louis Pradel, 59 boulevard Pinel, 69500 Hospices Civils de Lyon, Lyon, France; 2 Laboratoire CarMeN, Université Claude Bernard Lyon 1, Inserm U1060, Lyon, France; 3 Département de Médecine Périopératoire, Anesthésie-Réanimation, CHU de Clermont-Ferrand, Clermont-Ferrand, France; 4 Université Clermont Auvergne, CNRS; Inserm U1103, 63000 Clermont-Ferrand, France; 5 Service D’Anesthésie-Réanimation, Hôpital Timone, AP-HM, Marseille, France; 6 Service D’Anesthésie-Réanimation, Nouvel Hôpital Civil, Hôpitaux Universitaires de Strasbourg, Strasbourg, France; 7 Université de Strasbourg, Strasbourg, France; 8 Département D’Anesthésie-Réanimation, CHRU de La Cavale Blanche, Brest, France; 9 Université de Bretagne Occidentale, Brest, France; 10 Service de Chirurgie Digestive, Groupe Hospitalier Paris Saint-Joseph, Paris, France; 11 Département d’Anesthésie-Réanimation, Hôpital Lariboisière, DMU PARABOL, AP-HP Nord et Université de Paris, Paris, France; 12 UMR-S 942, Inserm, Paris, France; 13 Pôle d’Anesthésie-Réanimation, CHU Lille, Univ. Lille, ULR 2694–METRICS, Lille, France; 14 Pôle d’Anesthésie-Réanimation, Hôpital Pellegrin, CHU de Bordeaux, Bordeaux, France; 15 Université de Bordeaux, France, Inserm 1034, Pessac, France; 16 Service d’Anesthésie-Réanimation Chirurgicale, Pôle Anesthésie Réanimations, Hôtel-Dieu, CHU de Nantes, Nantes, France; 17 Université de Nantes, Nantes, France; 18 Service d’Anesthésie-Réanimation, Hôpital Européen Georges Pompidou, AP-HP, Paris, France; 19 Université de Paris, Paris, France; 20 Inserm UMR S1140, Paris, France; 21 Département d’Anesthésie-Réanimation, Hôpital Bichat Claude Bernard, AP-HP Nord, Paris, France; 22 Université de Paris, Paris, France

**Keywords:** Hemodynamic optimization, Blood pressure, Fluid responsiveness, Vasopressors, Perioperative morbidity, High-risk surgery, Health costs

## Abstract

Despite a large body of evidence, the implementation of guidelines on hemodynamic optimization and goal-directed therapy remains limited in daily routine practice. To facilitate/accelerate this implementation, a panel of experts in the field proposes an approach based on six relevant questions/answers that are frequently mentioned by clinicians, using a critical appraisal of the literature and a modified Delphi process. The mean arterial pressure is a major determinant of organ perfusion, so that the authors unanimously recommend not to tolerate absolute values below 65 mmHg during surgery to reduce the risk of postoperative organ dysfunction. Despite well-identified limitations, the authors unanimously propose the use of dynamic indices to rationalize fluid therapy in a large number of patients undergoing non-cardiac surgery, pending the implementation of a “validity criteria checklist” before applying volume expansion. The authors recommend with a good agreement mini- or non-invasive stroke volume/cardiac output monitoring in moderate to high-risk surgical patients to optimize fluid therapy on an individual basis and avoid volume overload. The authors propose to use fluids and vasoconstrictors in combination to achieve optimal blood flow and maintain perfusion pressure above the thresholds considered at risk. Although purchase of disposable sensors and stand-alone monitors will result in additional costs, the authors unanimously acknowledge that there are data strongly suggesting this may be counterbalanced by a sustained reduction in postoperative morbidity and hospital lengths of stay. Beside existing guidelines, knowledge and explicit clinical reasoning tools followed by decision algorithms are mandatory to implement individualized hemodynamic optimization strategies and reduce postoperative morbidity and duration of hospital stay in high-risk surgical patients.

## Introduction

Postoperative mortality has been reported to vary between 1 and 4% in developed countries [1]. This mortality rate is orders of magnitude higher than that of anesthesia-related deaths, even for patients with severe comorbidities, and explains the renewed interest on strategies that would result into lower postoperative mortality. Hemodynamic optimization (goal-directed therapy or GDT) has been recommended by several international guidelines because it has been shown consistently to improve outcomes [2]. However, the implementation of guidelines on GDT remains limited in routine practice [3, 4]. The explanations for absent/poor implementation of the guidelines are numerous [5–7] but it is possible that the current guidelines on perioperative GDT are on the one hand not explicit enough to allow a reproducible decision-making process, and on the other hand not suited to manage uncertainty [8]. Management of uncertainty refers to situations where there is no evidence reported in the guidelines or situations where the clinical reasoning tools (analysis of “abnormality”, critical analysis of the information gathered from the clinical situations/monitors, positive and differential diagnoses, treatment plan, evaluation of the effectiveness of the treatment plan) are not explicit [9].

To facilitate/accelerate implementation of guidelines on perioperative GDT, a national panel of experts in the field, representative of the whole French territory and who have previously collaborated on guidelines of the French Society of Anesthesiology and Critical Care is proposing an approach based on questions/answers on issues that are frequently mentioned by clinicians. These six relevant questions are:

1. Which blood pressure goals should be targeted during anesthesia and the perioperative period?
2. Intraoperative fluids management: restrictive, standard, liberal and beyond?
3. Are dynamic indices and maneuvers useful to predict and manage volume expansion?
4. When should we measure stroke volume (SV) and cardiac output (CO)?
5. Fluids or vasoconstrictors: how to decide?
6. What is the economic impact of hemodynamic monitoring for GDT?

The members of the panel first identified the questions and subsequently organized the answers by summarizing elements of guidelines, knowledge, and explicit clinical reasoning tools followed by decision algorithms when appropriate. The decision algorithms were adopted following a modified Delphi process and the RAND/UCLA Appropriateness Method [10]. We consider that explicit clinical reasoning tools will improve the decision-making process and facilitate management of uncertainty.

## Q1. Which blood pressure goals should be targeted during anesthesia and the perioperative period?

Monitoring blood pressure is a prerequisite during anesthesia. The main objective is to prevent postoperative complications resulting from either arterial hypo- or hypertension. The arterial pressure can be monitored at different anatomical sites, using various techniques, non-invasively or invasively, and in a continuous or intermittent manner. Although all excessive excursions of arterial pressure may be detrimental, the association between intraoperative arterial hypotension and postoperative complications is the most thoroughly documented.

To date, there is no universal definition for arterial hypotension during anesthesia. However, evidence from several large observational studies suggest that intraoperative mean arterial pressure (MAP) below 60–70 mmHg may be associated with postoperative acute kidney injury, myocardial injury, and death [11–14]. Although injury is magnified with increasing hypotension magnitude, available evidence suggests that MAP below 60 mmHg sustained for 5 min or more may be associated with organ dysfunction and increased mortality [15, 16]. Elevated risks or organ injury were also reported with prolonged exposure (< 10 min) to MAP below 70 mmHg [17]. To specifically prevent acute kidney injury, French Guidelines suggest to maintain MAP between 60 and 70 mmHg intraoperatively [18]. In patients with chronic arterial hypertension undergoing elective non-cardiac surgery, targeting MAP values higher than 70 mmHg may be reasonable, ideally adapted to the clinical and surgery conditions [19]. Even if systolic, pulse pressure (the difference between systolic and diastolic pressures) and MAP were recently found to have comparable discriminative ability in evaluating the risk of organ injury [20], MAP is the major determinant of organ perfusion. Moreover, systolic and diastolic pressures are indirectly calculated from MAP and may be less reliable when the oscillometric method is used. For this reason, MAP should be the main monitoring variable on which interventions to prevent/correct arterial hypotension should be based on. Whether or not relative changes in MAP for a given patient rather than absolute values should be preferred is still a matter of debate. In a simpler approach, anesthetic management of arterial hypotension could be based on absolute values without considering percentage changes from (difficult to document) preoperative arterial pressure values [21]. In a more complex approach, a multicenter randomized controlled trial performed in high-risk surgical patients undergoing major abdominal surgery (all received SV-guided intraoperative fluid) compared an individualized strategy using low-dose norepinephrine to maintain intraoperative systolic arterial pressure within 10% of the preoperative reference value to a strategy of standard management. The study revealed a significant reduction in postoperative organ dysfunction in the individualized strategy group [22]. Of note, the use of intraoperative continuous low-dose norepinephrine on a devoted peripheral intravenous line was safe and can be recommended for routine practice.

Finally, because even short cumulative durations of arterial hypotension are associated with poor outcome and because continuous (*versus* intermittent) measurement of arterial pressure was associated with higher sensitivity to diagnose arterial hypotension during anesthesia, continuous measurement of arterial pressure should be preferred. Non-invasive continuous monitoring of arterial pressure is not yet considered interchangeable with invasive monitoring and there is insufficient evidence to recommend its use in high-risk surgical patients and/or high-risk surgery. Future efforts should concentrate on trying to verify whether treating hypotensive episodes as detected by those new techniques results in improvement in patient outcome rather that repeating validation studies that are bound to provide the same results over and over.

**Experts’ opinion:** ***We propose to prefer continuous invasive arterial pressure monitoring in moderate to high-risk surgical patients, ideally using an algorithm-based approach which aims at preventing/managing arterial hypotension. We also propose to maintain MAP above 65 mmHg or within 10–20% of preoperative reference resting values (agreement 100%).***

## Q2. Intraoperative fluids management: restrictive, standard, liberal and beyond?

Intravenous fluid administration is the most frequent therapeutic intervention to maintain or restore tissue perfusion during surgical procedures. However, it is well established that inadequate volume therapy can result in deleterious effects, especially in frail or high-risk patients. Insufficient fluid administration will lead to reduced flow and potentially inadequate perfusion in some territories where the conductive vessels are more resistive. These territories may differ from one patient to another and can involve every organ. The reduced local blood flow can induce cellular hypoxia and subsequent organ dysfunction or failure. On the other hand, if fluid is administered in excess, venous congestion and edema will ensue. The consequences of fluids in excess are probably as deleterious as the consequences of hypovolemia, and many studies have established a clear relation between positive fluid balance and postoperative complications [23, 24]. Thus, targeting the right amount of volume expander required by each surgical patient during the procedure is a daily challenge for practitioners. Recommendations are often blurry with statements indicating to maintain “adequate volemia” or “optimal volume”, which do not translate into quantitative meaningful information. Even worse, some protocols suggest to administer an identical predefined amount of fluids to everyone, taking into account the body weight and the duration of the surgical procedure, assuming that the requirements and the tolerance are similar for all individuals. This “one size fits all” strategy is bound to result in inadequate volume management in the vast majority of patients [25]. Low-risk patients will usually tolerate the deviation from their adequate volume requirements, but high-risk patients will be exposed to the above-mentioned complications. The available literature do not provide evidence-based recommendations regarding continuous fluid infusion. The basal fluid losses via insensible perspiration are approximately 0.5 ml/kg/h, extending to 1 ml/kg/h during major abdominal surgery [26]. When continuous fluid infusion is used, it should be limited to less than 2 ml/kg/h, including drug infusion [25].

**Fig. 1:**
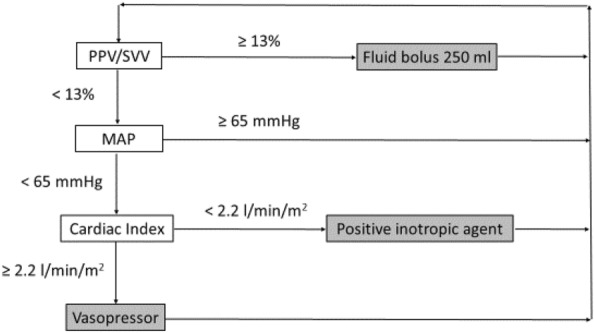
Typical intraoperative goal-directed therapy algorithm based on an individualized approach. *MAP* mean arterial pressure, *PPV* pulse pressure variation, *SVV* stroke volume variation. Values for PPV/SVV, MAP and cardiac index are indicative and must be adapted on an individual basis. The use of vasopressors could also be considered when diastolic arterial pressure < 40 mmHg

Therefore, optimizing tissue perfusion in high-risk patients relies on an individualized approach. The MAP will be maintained above a level close to the usual value of the patient, as suggested above. Minimal value for hemoglobin concentration and transfusion thresholds will vary according to the comorbidities of each patient. And since the ideal values of organ flow for a given patient are unknown, it is recommended to titrate fluids using small iterative boluses (100 to 250 ml crystalloids over 5 to 10 min) guided by measurements of SV variation [27] (Fig. 1). A SV increase > 10–12% (to avoid being confounded by measurement variability) assessed one minute after the end of fluid infusion indicates that the patient is able to increase flow and tissue perfusion in response to fluids. A lack of increase of SV after fluid bolus is the most reliable indication that additional volume therapy may generate congestion and edema and thus become deleterious. The smaller the volume administered, the minimal the congestion resulting from the unnecessary volume overload. The number of studies that have demonstrated the reduction in complications associated with a SV-guided fluid titration in high-risk surgical patients provides compelling evidence to support that simple approach to minimize the deleterious side effects of intraoperative fluids [28, 29].

**Experts’ opinion:** ***We propose to routinely use a personalized approach of intraoperative fluid infusion/volume expansion based on the individual hemodynamic response to volume titration to reduce the deleterious side effects of fluids and improve patients’ outcome (agreement 100%).***

## Q3. Are dynamic indices and maneuvers useful to predict and manage volume expansion?

Fluid responsiveness is defined as a significant increase in blood flow in response to a fluid bolus [30]. Predicting fluid responsiveness is useful to identify patients who may benefit from volume expansion and, more importantly, to prevent fluid administration in non-responders [31]. Cardiac preload indices, such as central venous pressure, have repeatedly been shown to be unreliable predictors of fluid responsiveness. On the contrary, the arterial pulse pressure variation (PPV) induced by mechanical ventilation is known as a sensitive and specific marker of fluid responsiveness [32]. In patients receiving controlled mechanical ventilation with a tidal volume ≥ 7–8 ml/kg of ideal body weight (IBW), fluid responsiveness is very likely when PPV is > 13%, very unlikely when PPV is < 9% and uncertain when PPV ranges between 9 and 13% (grey zone of uncertainty) [33]. Importantly, tidal volumes of 7–8 ml/kg of IBW are consistent with recent recommendations for perioperative lung-protective ventilation [34]. Since the sentinel study by Lopes et al. [35], numerous clinical studies using either PPV or the pulse contour-derived stroke volume variation (SVV) to individualize fluid therapy showed a decrease in postoperative complications and hospital lengths of stay [36]. The estimation of PPV requires either invasive or non-invasive recording of a continuous arterial pressure waveform [37–39]. There are several limitations to the use of PPV/SVV which have been described in detail elsewhere [40]. For instance, protective mechanical ventilation is a potential obstacle to the use of PPV when very low tidal volumes are used (e.g. 6 ml/kg IBW or less) [41]. During very low tidal volume ventilation, a high PPV still suggests fluid responsiveness whereas a low PPV cannot rule out fluid responsiveness. Therefore, alternative methods have been proposed to predict fluid responsiveness. They include the assessment of PPV changes during a tidal volume challenge or during a mini-fluid challenge (Table 1). Thus, a 3.5% absolute increase in PPV during a transient rise in tidal volume from 6 to 8 ml/kg IBW could be useful to predict fluid responsiveness with sensitivity and specificity values > 90% [42]. As well, an absolute decrease in PPV > 2% during a mini-fluid challenge could help to predict fluid responsiveness [43]. Limitations to the interpretation of PPV also include cardiac arrhythmias, right and left ventricular failure, decreased lung compliance, and spontaneous breathing activity [32, 40]. Importantly, the coelioscopic surgery-induced pneumoperitoneum is a frequent situation which decreases thoracic compliance, leading to changes in the interpretation of PPV (a situation defined as false positive but this requires a commentary: the patient is preload-dependent due to decreased venous return secondary to increased abdominal pressure meaning that volume expansion will unfrequently correct fluid responsiveness) [44, 45]. Further studies are needed to determine the impact of an increased abdominal pressure between 10 and 15 mmHg on PPV threshold values that should be considered to identify intraoperative fluid responsiveness. Meanwhile, PPV must be interpreted with caution in that specific surgical setting or in patients in prone position. Finally, dynamic indices seem to predict fluid responsiveness with insufficient accuracy in an open-chest condition during cardiac and/or thoracic surgery [46].

**Table 1:** Main advantages and limitations of dynamic indices and maneuvers developed to predict fluid responsiveness (chronologic order from top to bottom)

Methods (year of first validation)	Main advantages	Main limitations
PPV (2000)	Automatically calculated by most bedside monitors	Need for general anesthesia, an arterial line and a tidal volume > 7 ml/kg
SVV (2001)	Automatically calculated by most CO monitors	Need for a CO monitor, general anesthesia, an arterial line and a tidal volume > 7 ml/kg
Changes in CO during a PLR maneuver (2006)	Useful when Vt < 7 ml/kg IBW	Need for a CO monitor, PLR maneuver difficult to perform during surgery
PVI (2008)	Non-invasive from a pulse ox	Need for general anesthesia and a tidal volume > 7 ml/kg, influenced by peripheral perfusion
Changes in SV during an EEO test (2009)	Useful when Vt < 7 ml/kg IBW	Need for a CO monitor, prone to error measurements (small magnitude of changes in SV)
Changes in SV during a mini-fluid challenge (2011)	Useful when Vt < 7 ml/kg IBW	Need for a CO monitor, prone to error measurements (small magnitude of changes in SV)
Changes in PPV during a mini-fluid challenge (2015)	Useful when Vt < 7 ml/kg IBW	Prone to error measurements (small magnitude of changes in PPV)
Changes in PPV during a Vt challenge (2017)	Useful when Vt < 7 ml/kg IBW	Prone to error measurements (small magnitude of changes in PPV)
Changes in SV during a LRM (2017)	Useful when Vt < 7 ml/kg IBW	Need for a CO monitor
Changes in PI during a PLR maneuver (2019)	Useful when Vt < 7 ml/kg IBW, non-invasive (pulse ox)	Influenced by peripheral perfusion, PLR maneuver difficult to perform during surgery Low level of scientific validation
Changes in PI during a LRM (2020)	Useful when Vt < 7 ml/kg IBW, non-invasive (pulse ox)	Influenced by peripheral perfusion Low level of scientific validation

*CO* cardiac output, *EEO* end-expiratory occlusion, *IBW* ideal body weight, *LRM* lung recruitment maneuver, *PI* perfusion index, *PLR* passive leg raising, *PPV* pulse pressure variation, *Pulse ox* pulse oximetry, *PVI* pleth variability index, *SV* stroke volume, *SVV* stroke volume variation, *Vt* tidal volume

Subsequently, it could be reasonable for routine practice to implement a “validity criteria checklist” before using PPV or similar approaches to estimate fluid responsiveness (Table 2) [47]. When PPV cannot be used, it remains possible to assess fluid responsiveness in surgical patients undergoing general anesthesia by measuring changes in SV during an end-expiratory occlusion test, a lung recruitment maneuver or during a mini-fluid challenge [48, 49]. Out of the operating room, the most validated maneuver is the passive leg raising test [50]. The main limiting factor to the clinical adoption of those methods is the availability of a cardiac output monitor to quantify SV changes (Table 1). The pleth variability index (PVI), a non-invasive surrogate for PPV, may also be useful to predict fluid responsiveness during surgery [51]. Recently, the quantification of changes in the peripheral perfusion index (PI), a variable used as a signal quality indicator by most pulse oximeters, has been proposed in an exploratory study to predict fluid responsiveness with acceptable sensitivity and specificity [52]. However, monitors using finger cuff technologies have in common the risk of poor reliability in cases of peripheral hypoperfusion.

**Table 2:** “Validity criteria checklist” before performing volume expansion based on pulse pressure variation/similar methods-provided information. Adapted from [47]

1	Is the patient ventilated with CMV without spontaneous efforts?
2	Is the patient ventilated with tidal volume at least 7–8 ml/kg IBW?
3	Is the patient in closed-chest condition?
4	Is the patient in sinus rhythm?
5	Is lung compliance normal?
6	Is the patient unaffected by valvular disease?
7	Is the patient unaffected by right/left ventricular dysfunction?
8	Does the patient have normal abdominal pressure?
9	Is the HR/RR ratio ≥ 3.6?
10	Can you safely assess the efficacy of VE without HR or vasomotor tone changes?

CMV: controlled mechanical ventilation; HR/RR: heart rate/respiratory rate ratio; IBW: ideal body weight; VE: volume expansion

**Experts’ opinion:** ***We propose to implement a “validity criteria checklist” before using PPV (or similar methods) to estimate fluid responsiveness, then to give iterative small fluid boluses to maintain intraoperative PPV (or similar methods) below the threshold value that defines fluid responsiveness (agreement 100%).***

## Q4. When should we measure stroke volume and cardiac output?

While guidelines highly recommend the assessment of CO/SV for perioperative fluid GDT in high-risk surgical patients [27], less than one-third of patients in Europe and in the United States actually benefit from CO monitoring during the perioperative period [53]. The main reasons reported by physicians are: (i) CO monitoring reference methods are too invasive; (ii) Non-invasive CO monitoring is unreliable; (iii) CO monitoring is useless to guide fluid optimization. Beyond classical knowledge regarding accuracy and precision of CO/SV measurements, other criteria should now be considered to implement a strategy of GDT in high-risk patients. These criteria are the mini-invasive or non-invasive approach of the monitoring method; instantaneous and continuous information; automatized assessment without external calibration; easy-to-use plug and play system; the absence of both operator-dependence and learning curve; reasonable cost; the ability to impact decision-making process and outcomes. Thus, many devices using various technologies have been commercially developed and scientifically evaluated during the last 20 years (Table 3). Those devices are undoubtedly not interchangeable with reference methods (namely Fick principle, bolus thermodilution and echocardiography). However, it remains difficult to give a universal definition of what is actually an acceptable agreement between a new method of CO measurement and a reference method [54]. Moreover, numerous studies and meta-analyses suggest that mini-invasive technologies such as esophageal Doppler [28] or pulse contour analysis [55] are useful to guide GDT and improve outcomes in high-risk surgical patients when compared with a standard of care. Calibrated or uncalibrated pulse contour analysis methods especially seem to have the favor of practitioners, being used in nearly 75% of cases [56, 57]. Thus, beside reference methods of CO measurement, often invasive, uneasy to implement and clearly underused in the operating room, new mini-invasive and non-invasive technologies, while not interchangeable with the latter, could be useful to facilitate implementation of GDT and should probably be employed to improve outcomes in moderate to high-risk surgical patients. Invasive reference methods are, however, sometimes unavoidable, as in cardiac surgery or shock states for instance. Clinicians should actually be aware that whatever the precision of the method used to estimate SV, the most important is to stop fluid administration as soon as SV no longer increase in response to fluid boluses. The majority of monitors used to estimate SV should be able to provide a clinically relevant answer to that crucial issue.

**Table 3:** Invasive, mini-invasive, and non-invasive cardiac output monitoring technologies

Device	Technology	Properties	Calibration
Cardio-Q *(Gamida)*	Esophageal Doppler	Mini-invasive, continuous, operator-dependent	No
NICO *(Novametrix)*	Fick principle applied to CO_2_	Non-invasive, discontinuous, operator-independent	No
PiCCO *(Getinge)* Volume View *(Edwards)*	Transpulmonary thermodilution	Invasive, discontinuous, operator-independent	Yes
Niccomo *(Imedex)* Physioflow *(Manatec)* ECOM *(ConMed)*	Bioimpedance	Non-invasive, continuous, operator-independent	No
NICOM *(Baxter)*	Bioreactance	Non-invasive, continuous, operator-independent	No
Vigileo/Flotrac *(Edwards)* Pulsioflex/ProAQT *(Getinge)* MostCare *(Vygon)* LIDCOrapid *(LIDCO Ltd)*	Pulse contour analysis	Mini-invasive, continuous, operator-independent	Optional
ClearSight *(Edwards)* NICCI *(Getinge)*	Digital photoplethysmography	Non-invasive, continuous, operator-independent	No
esCCO *(Nihon Kohden)*	Pulse wave transit time	Non-invasive, continuous, operator-independent	Optional

**Experts’ opinion:** ***We propose to use mini- or non-invasive continuous methods to monitor CO/SV rather than invasive reference techniques to implement perioperative GDT in moderate to high-risk patients undergoing non-cardiac surgery (agreement 83%).***

*Two experts pointed out problematic limits regarding accuracy of those mini- or non-invasive methods in patients with hypothermia or peripheral hypoperfusion or receiving continuous infusion of vasopressors.*

## Q5. Fluids or vasoconstrictors: how to decide?

Tissue perfusion means delivering enough oxygen, glucose and other metabolites to every cell, as well as clearing the byproducts of cell metabolism. This is achieved when the flow at the level of the microcirculation of each organ is “adequate”, a condition that is impossible to assess with our current monitoring capabilities. Therefore, we concentrate our attention on the major determinants that will most likely result in adequate microcirculatory blood flow: MAP value that is sufficient to drive blood flow through every organ and a systemic blood flow that is able to cover for the total body oxygen requirements. Thus, ideally, both instantaneous arterial pressure and beat by beat systemic blood flow should be monitored in addition to heart rate to “optimize” tissue perfusion. Diastolic arterial pressure is mainly determined by vascular tone [58]. Low diastolic arterial pressure could be likened to as a sign of vasodilation (by lowering vascular tone) and it has been proposed to introduce vasopressors in ICU patients when diastolic arterial pressure is below 40 mmHg [59]. However, available data are conflicting in the perioperative setting [20]. When tissue perfusion is deemed inadequate, or when its “optimization” is attempted prophylactically in a high-risk patient, the first therapeutic approach is usually based on intravenous fluid titration, as developed above. In the meantime, if arterial pressure values are considered too low with respect to the “usual values” of the patient, vasoconstrictors may be administered intravenously as small iterative boluses or as a continuous infusion to achieve the personalized pressure value considered adequate [22, 60] (Fig. 1).

Despite the fact that anesthesia induction produces vasodilation and reduces venous return via a reduction in effective volemia or stressed volume (as a consequence of the increased venous capacitance without any reduction in intravascular volume), physicians restore systemic flow by giving intravenous fluids rather than vasoconstrictors. This approach is empirical and does not seem to respond to the primary determinant of reduced venous return, which is increased venous compliance. Rather, it is now established that optimization of both flow and pressure is associated with improved outcomes in high-risk patients (Table 4). Fluids and vasoconstrictors must be used in combination to achieve optimal blood flow and maintain pressure above the thresholds considered “at risk”. Achieving optimal flow while avoiding venous congestion cannot be done without the guidance of SV monitoring. Continuous measurement of arterial pressure is the mandatory counterpart for high-risk patients in whom even brief transient arterial hypotension can generate adverse events. The incorporation in our monitoring devices of artificial intelligence and machine-learning algorithms trained to detect changes in hemodynamics that precede clinically apparent hypotension offers interesting perspectives [65]. Trials are currently ongoing to confirm that this technology can effectively reduce hypotension during surgery and further improve patient outcome [66].

**Table 4:** The impact of hemodynamic optimization on postoperative morbidity: results from five meta-analyses in high-risk surgical patients

References	Number of studies (patients)	Reduction in postoperative morbidity (%)
Hamilton [61]	29 (4,805)	57
Grocott [62]	31 (5,292)	32
Pearse [63]	22 (3,024)	23
Michard [55] ^a^	19 (2,159)	54
Chong [64]	95 (11,659)	34

^a^Non-calibrated pulse contour analysis only

The main vasopressors used in the operating theatre are phenylephrine, ephedrine, and norepinephrine. Their pharmacological effects are somewhat different but there are no data on the superiority of one vasopressor over the others. Phenylephrine could, however, be responsible for a significant decrease in cardiac output and organ perfusion, especially the brain [67] and in patients without preload dependence [68].

**Experts’ opinion:** ***We propose to use fluids and vasoconstrictors in combination via a GDT algorithm to simultaneously achieve perioperative optimal blood flow and perfusion pressure, avoid volume overload and improve outcome in high-risk surgery patients (agreement 100%).***

## Q6. What is the economic impact of hemodynamic monitoring for GDT?

Implementation of GDT and its additional hemodynamic monitoring has a cost. This increased cost could be perceived as an obstacle to hospital adoption. The cost of hemodynamic monitoring is highly variable, not only from one technique to another, but also from one country to the other, depending on reimbursement policies. Whatever such country-dependent pricing policies of monitoring devices, there is a common denominator: postoperative complications dramatically increase hospital costs. Studies have shown that the average cost difference between a patient with one or more complications and a patient without any complication ranges between 10,000€ and 30,000€ [69, 70]. We personally found in 227 patients undergoing colorectal surgery and scheduled for an ERAS program including GDT that the occurrence of a complication had a mean extra-cost of 3,167€ (additional treatment cost ≈1,030€ and prolonged length of stay ≈2,058€) which was not covered by the average 2,777€ additional public health system complication reimbursement. The largest economic evaluation published so far (> 700 high-risk patients undergoing major gastrointestinal surgery), done in the UK with a pulse contour technique, showed net savings around 400£/patient [71]. The reduction in postoperative morbidity is also often associated with a reduction in hospital length of stay [72]. In that case, the increase in the number of free beds may allow a boost in surgical activity, a decreased wait times for patients, and increased revenue.

The « MERCI» equation [73] enables an easy estimation of the possible Investment (I) to implement hemodynamic monitoring at no net costs. It takes into account the current morbidity rate (M), the expected reduction (ER) in postoperative morbidity, and the current cost (C) of complications:

$${\text{M }} \times {\text{ ER }} \times {\text{ C }} = {\text{ I}}{.}$$

As an example, if the morbidity rate after colorectal surgery is 25% (M = 25%), the relative reduction in postoperative morbidity is 23% (ER = 23%), and the average cost of complications per patient is 15,000€, the investment to implement hemodynamic monitoring at no net cost is 863€/patient (0.25 × 0.23 × €15,000 = 863€). If the cost of monitoring is less than 863€/patient (which is most often the case), the difference would be savings to the health system. The higher the risk of postoperative complications and the cost of those complications, the more favorable the economic impact of GDT will be. Several studies have used the MERCI equation to predict the economic impact of hemodynamic monitoring in large populations of patients undergoing major non-cardiac surgery [69, 74, 75]. They reported possible investments of around 500–1000€/patient. Since it may end up being the main driver for adoption by hospital administration, the impact of hemodynamic monitoring on hospital profitability should be well known by physicians arguing for GDT in their institutions.

**Experts’ opinion:** ***We propose that clinicians explain to national/hospital decision-makers that the extra-cost due to hemodynamic monitoring when implementing a perioperative GDT strategy is counterbalanced by the reduction in postoperative complications and hospital length of stay in high-risk surgery. This could be estimated at each institution level using the MERCI equation (agreement 100%).***

## Conclusion

Because the current guidelines on perioperative hemodynamic optimization are not explicit enough to allow a reproducible decision-making process and also not suited to manage uncertainty, their implementation remains limited in routine practice. Numerous relevant questions are frequently asked by practitioners who need explicit clinical reasoning tools and treatment plans to develop strategies of blood pressure and cardiac output monitoring and optimization on an individual basis and at a reasonable cost.

## Data Availability

Not applicable.
